# Bis(hydrogen l-glutamato)palladium(II)

**DOI:** 10.1107/S1600536811035860

**Published:** 2011-09-14

**Authors:** Antje Seifert, Christoph Wagner, Kurt Merzweiler

**Affiliations:** a Institut für Chemie, Naturwissenschaftliche Fakultät II, Martin-Luther-Universität Halle-Wittenberg, Kurt-Mothes-Str. 2, 06120 Halle, Germany

## Abstract

In the title compound, [Pd(C_5_H_8_NO_4_)_2_], the Pd(II) atom is coordinated by two O atoms and two N atoms of two *N,O*-chelating hydrogen l-glutatmate ligands in a square–planar geometry with the N and O atoms in a mutually *trans* arrangement. The complex units are embedded in a network of N—H⋯O and O—H⋯O hydrogen-bonding inter­actions that stabilize the three-dimensional crystal structure. The strongest hydrogen bonds are formed between the γ-COOH untis of adjacent glutamate ligands, leading to dimers of the type *R*_2_^2^(8) with O⋯O separations of 2.640 (6) Å.

## Related literature

For the synthesis of the title compound, see: Spacu & Scherzer (1962 ▶). For the structures of related palladium complexes with amino acid ligands, see: Vagg (1979 ▶); Jarzab *et al.* (1973 ▶); Sabat *et al.* (1979 ▶); Pletnev *et al.* (1992 ▶); Hao *et al.* (2007 ▶); Gao *et al.* (2008 ▶).
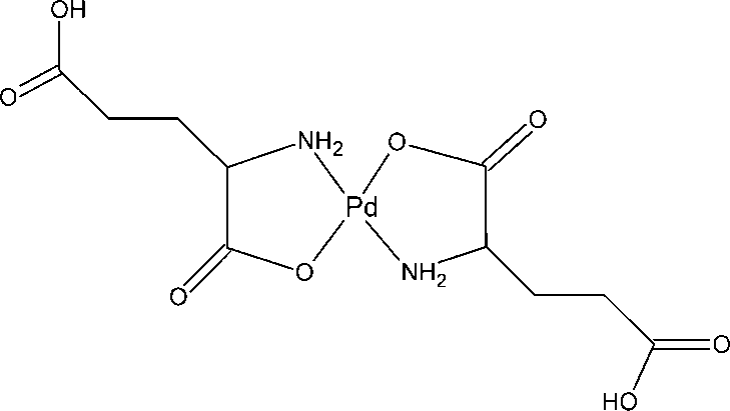


## Experimental

### 

#### Crystal data

[Pd(C_5_H_8_NO_4_)_2_]
*M* *_r_* = 398.66
Triclinic, 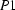

*a* = 4.8858 (3) Å
*b* = 5.1605 (4) Å
*c* = 13.3651 (9) Å
α = 93.725 (6)°
β = 99.734 (6)°
γ = 104.245 (6)°
*V* = 319.90 (4) Å^3^
*Z* = 1
Mo *K*α radiation
μ = 1.49 mm^−1^
*T* = 200 K
0.17 × 0.06 × 0.04 mm

#### Data collection

Stoe IPDS 2T diffractometer
Absorption correction: integration (*X-RED*; Stoe & Cie, 2009 ▶) *T*_min_ = 0.806, *T*_max_ = 0.973
4724 measured reflections
2406 independent reflections
2389 reflections with *I* > 2σ(*I*)
*R*_int_ = 0.044

#### Refinement

*R*[*F* ^2^ > 2σ(*F* ^2^)] = 0.029
*wR*(*F* ^2^) = 0.066
*S* = 1.04
2406 reflections
208 parameters
9 restraints
H atoms treated by a mixture of independent and constrained refinement
Δρ_max_ = 0.37 e Å^−3^
Δρ_min_ = −0.74 e Å^−3^
Absolute structure: Flack (1983 ▶), 1146 Friedel pairs
Flack parameter: −0.02 (4)

### 

Data collection: *X-AREA* (Stoe & Cie, 2009 ▶); cell refinement: *X-AREA*; data reduction: *X-RED* (Stoe & Cie, 2009 ▶); program(s) used to solve structure: *SHELXS97* (Sheldrick, 2008 ▶); program(s) used to refine structure: *SHELXL97* (Sheldrick, 2008 ▶); molecular graphics: *DIAMOND* (Brandenburg, 2009 ▶); software used to prepare material for publication: *SHELXL97* and *PLATON* (Spek, 2009 ▶).

## Supplementary Material

Crystal structure: contains datablock(s) I, publication_text. DOI: 10.1107/S1600536811035860/wm2526sup1.cif

Structure factors: contains datablock(s) I. DOI: 10.1107/S1600536811035860/wm2526Isup2.hkl

Additional supplementary materials:  crystallographic information; 3D view; checkCIF report

## Figures and Tables

**Table 1:** Selected bond lengths (Å)

Pd—N1	2.072 (9)
Pd—N2	2.005 (11)
Pd—O1	1.976 (8)
Pd—O5	2.024 (7)

**Table 2:** Hydrogen-bond geometry (Å, °)

*D*—H⋯*A*	*D*—H	H⋯*A*	*D*⋯*A*	*D*—H⋯*A*
N2—H9⋯O6^i^	0.85 (2)	2.15 (4)	2.979 (15)	162 (8)
N2—H10⋯O5^ii^	0.85 (2)	2.43 (8)	3.121 (13)	138 (10)
O7—H16⋯O4^iii^	0.85 (2)	1.80 (2)	2.639 (6)	169 (7)
O3—H8⋯O8^iv^	0.86 (2)	1.79 (3)	2.640 (6)	167 (7)
N1—H1⋯O2^v^	0.85 (2)	2.15 (3)	2.996 (15)	170 (9)
N1—H2⋯O1^vi^	0.85 (2)	2.30 (8)	2.998 (13)	139 (10)
N1—H2⋯O5^vii^	0.85 (2)	2.42 (7)	3.117 (13)	140 (10)

Symmetry codes: (i) 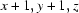; (ii) 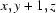; (iii) 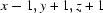; (iv) 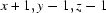; (v) 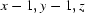; (vi) 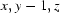; (vii) 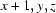.

**Fig. 1.**
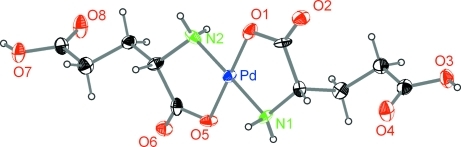
Molecular structure of (I). Displacement ellipsoids are drawn at the 50% probabiltiy level.

**Fig. 2.**
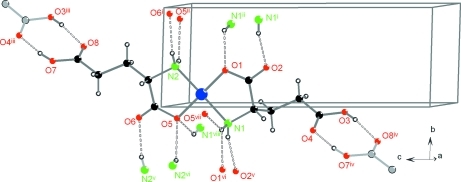
Arrangement of the hydrogen bonds around the complex unit. [Symmetry operators: (i) x + 1, y + 1, z; (ii) x, y + 1, z; (iii) x - 1, y + 1, z + 1; (iv) x + 1, y - 1, z - 1; (v) x - 1, y - 1, z; (vi) x, y - 1, z; (vii) x + 1, y, z; (viii) x - 1, y, z.]

## supplementary crystallographic information

### Comment

The title compound, [Pd(C_5_H_8_NO_4_)_2_] (I), consists of a palladium(II) atom which is coordinated by two N and two O atoms of two chelating hydrogen glutamate ligands. The metal atom adopts a distorted square–planar coordination with the two N atoms and the two O atoms in mutual *trans* arrangements. The maximum deviation from the least squares plane through the atoms Pd, N1, N2, O1 and O5 is 0.026 Å for N2. The distances Pd—O (1.976 (8), 2.024 (7) Å) and Pd—N (2.005 (11), 2.072 (9) Å) are in agreement with the values observed for other Pd(II) amino acid derivatives, like *cis*-bis(*L*-tyrosinato)-palladium(II) hemihydrate (Jarzab *et al.*, 1973), bis(*L*-tyrosinato)-palladium(II) (Sabat *et al.*, 1979), bis(*L*-valinato)-palladium(II) monohydrate (Pletnev *et al.*, 1992); Hao *et al.*, 2007), *cis*-bis(*L*-aspartato-*N,O*)-palladium(II) (Gao *et al.*, 2008) or bis(*L*-serinato)-palladium(II) (Vagg, 1979). For both the hydrogen glutatmate ligands, the five membered PdNC_2_O chelate rings adopt envelope conformations with a nearly coplanar arrangement of the Pd, O, C and N atoms at the flap positions.

The main conformational difference between both chelate rings arises from the orientation of the carboxyethyl groups. In the case of C2 the carboxyethyl group is in an axial position and for C7 an equatorially arrangend carboxyethyl group is observed (Fig. 1).

The packing of the complex units is supported by different types of hydrogen bonding interactions. The strongest hydrogen bridges are formed between the γ-carboxyl groups of neighbouring molecules. Consequently, *R*_2_^2^(8) motifs are observed and the complex units are arranged in chains. Additionally, there are N—H···O hydrogen bridges of the type C_1_^1^(4) and C_1_^1^(5) which are formed between amino groups and oxygen atoms of adjacent α-carboxylate groups (Fig. 2)

### Experimental

The title compound was prepared from K_2_PdCl_4_ and sodium hydrogen glutamate according to a procedure described by Spacu & Scherzer (1962). For the growth of single crystals, the reaction mixture was stored at 278 K for several weeks.

### Refinement

C-bound H atoms of the hydrogen glutamate units were positioned geometrically and refinded using a riding model with *U*_iso_(H) = 1.2 *U*_eq_(C) [*d*(C—H) = 0.99 (for C–H) and 1.00 Å (for CH_2_)]. H atoms attached to N and O were located from difference fourier maps and refined with N—H distances fixed at 0.85 (2) Å (*U*_iso_(H) = 1.2 *U*_eq_(N)) and O—H distances fixed at 0.85 (2) and 0.86 (2) Å (*U*_iso_(H) = 1.2 *U*_eq_(O)).

### Crystal data

**Table:**

[Pd(C_5_H_8_NO_4_)_2_]	*Z* = 1
*M**_r_* = 398.66	*F*(000) = 201
Triclinic, *P*1	*D*_x_ = 2.070 Mg m^−^^3^
Hall symbol: P 1	Mo *K*α radiation, λ = 0.71073 Å
*a* = 4.8858 (3) Å	Cell parameters from 7673 reflections
*b* = 5.1605 (4) Å	θ = 3.1–29.3°
*c* = 13.3651 (9) Å	µ = 1.49 mm^−^^1^
α = 93.725 (6)°	*T* = 200 K
β = 99.734 (6)°	Prism, yellow
γ = 104.245 (6)°	0.17 × 0.06 × 0.04 mm
*V* = 319.90 (4) Å^3^	

### Data collection

**Table:**

Stoe IPDS 2T diffractometer	2406 independent reflections
Radiation source: sealed X-ray tube, 12 x 0.4 mm long-fine focus	2389 reflections with *I* > 2σ(*I*)
plane graphite	*R*_int_ = 0.044
Detector resolution: 6.67 pixels mm^-1^	θ_max_ = 26.0°, θ_min_ = 3.1°
ω–scans	*h* = −6→6
Absorption correction: integration (*X-RED*; Stoe & Cie, 2009)	*k* = −6→6
*T*_min_ = 0.806, *T*_max_ = 0.973	*l* = −16→16
4724 measured reflections	

### Refinement

**Table:**

Refinement on *F*^2^	Secondary atom site location: difference Fourier map
Least-squares matrix: full	Hydrogen site location: inferred from neighbouring sites
*R*[*F*^2^ > 2σ(*F*^2^)] = 0.029	H atoms treated by a mixture of independent and constrained refinement
*wR*(*F*^2^) = 0.066	*w* = 1/[σ^2^(*F*_o_^2^) + (0.0356*P*)^2^ + 0.1842*P*] where *P* = (*F*_o_^2^ + 2*F*_c_^2^)/3
*S* = 1.04	(Δ/σ)_max_ < 0.001
2406 reflections	Δρ_max_ = 0.37 e Å^−^^3^
208 parameters	Δρ_min_ = −0.74 e Å^−^^3^
9 restraints	Absolute structure: Flack (1983), 1146 Friedel pairs
Primary atom site location: structure-invariant direct methods	Flack parameter: −0.02 (4)

### Special details

**Table:**

Geometry. All e.s.d.'s (except the e.s.d. in the dihedral angle between two l.s. planes) are estimated using the full covariance matrix. The cell e.s.d.'s are taken into account individually in the estimation of e.s.d.'s in distances, angles and torsion angles; correlations between e.s.d.'s in cell parameters are only used when they are defined by crystal symmetry. An approximate (isotropic) treatment of cell e.s.d.'s is used for estimating e.s.d.'s involving l.s. planes.
Refinement. Refinement of *F*^2^ against ALL reflections. The weighted *R*-factor *wR* and goodness of fit *S* are based on *F*^2^, conventional *R*-factors *R* are based on *F*, with *F* set to zero for negative *F*^2^. The threshold expression of *F*^2^ > σ(*F*^2^) is used only for calculating *R*-factors(gt) *etc*. and is not relevant to the choice of reflections for refinement. *R*-factors based on *F*^2^ are statistically about twice as large as those based on *F*, and *R*- factors based on ALL data will be even larger.

### Fractional atomic coordinates and isotropic or equivalent isotropic displacement parameters (Å^2^)

**Table:**

	*x*	*y*	*z*	*U*_iso_*/*U*_eq_	
C1	0.649 (3)	0.240 (2)	0.7428 (9)	0.020 (3)	
C2	0.478 (2)	−0.043 (2)	0.7031 (8)	0.015 (2)	
H3	0.6076	−0.1464	0.6791	0.017*	
C3	0.2260 (11)	−0.0501 (11)	0.6139 (4)	0.0219 (11)	
H4	0.1138	−0.2395	0.5939	0.026*	
H5	0.0971	0.0487	0.6394	0.026*	
C4	0.3130 (13)	0.0694 (11)	0.5184 (4)	0.0234 (11)	
H6	0.1410	0.1014	0.4755	0.028*	
H7	0.4546	0.2460	0.5398	0.028*	
C5	0.4388 (11)	−0.1010 (11)	0.4554 (4)	0.0224 (11)	
C6	−0.125 (2)	0.009 (2)	1.0111 (8)	0.018 (2)	
C7	0.098 (2)	0.272 (2)	1.0603 (9)	0.019 (2)	
H11	0.2662	0.2244	1.1017	0.023*	
C8	−0.0210 (12)	0.4448 (10)	1.1299 (4)	0.0213 (11)	
H12	−0.2096	0.4604	1.0938	0.026*	
H13	0.1110	0.6279	1.1447	0.026*	
C9	−0.0580 (13)	0.3289 (13)	1.2308 (4)	0.0249 (12)	
H14	−0.1794	0.1414	1.2162	0.030*	
H15	0.1325	0.3254	1.2694	0.030*	
C10	−0.1944 (12)	0.4914 (11)	1.2950 (4)	0.0210 (11)	
N1	0.345 (2)	−0.179 (2)	0.7842 (7)	0.018 (2)	
H2	0.464 (16)	−0.25 (2)	0.820 (7)	0.022*	
H1	0.196 (12)	−0.311 (12)	0.767 (7)	0.022*	
N2	0.194 (2)	0.405 (2)	0.9716 (8)	0.020 (2)	
H10	0.054 (15)	0.46 (2)	0.944 (8)	0.024*	
H9	0.346 (13)	0.526 (13)	0.999 (7)	0.024*	
O1	0.5808 (17)	0.3460 (16)	0.8246 (6)	0.023 (2)	
O2	0.8325 (17)	0.3614 (16)	0.6976 (6)	0.0259 (16)	
O3	0.3977 (9)	−0.0697 (8)	0.3575 (3)	0.0278 (8)	
H8	0.503 (13)	−0.150 (12)	0.328 (5)	0.033*	
O4	0.5723 (10)	−0.2598 (10)	0.4909 (3)	0.0289 (9)	
O5	−0.0695 (16)	−0.1097 (14)	0.9320 (6)	0.0206 (18)	
O6	−0.3437 (17)	−0.0908 (16)	1.0453 (6)	0.0237 (15)	
O7	−0.1369 (9)	0.4718 (8)	1.3941 (3)	0.0267 (8)	
H16	−0.220 (15)	0.553 (12)	1.432 (5)	0.032*	
O8	−0.3538 (9)	0.6250 (9)	1.2573 (3)	0.0319 (9)	
Pd	0.26234 (18)	0.11739 (15)	0.87796 (9)	0.01627 (10)	

### Atomic displacement parameters (Å^2^)

**Table:**

	*U*^11^	*U*^22^	*U*^33^	*U*^12^	*U*^13^	*U*^23^
C1	0.021 (6)	0.011 (5)	0.029 (6)	0.004 (4)	0.005 (5)	−0.001 (4)
C2	0.007 (4)	0.025 (4)	0.014 (4)	0.007 (3)	0.004 (3)	0.003 (3)
C3	0.017 (2)	0.026 (3)	0.023 (3)	0.007 (2)	0.003 (2)	0.000 (2)
C4	0.027 (3)	0.024 (3)	0.021 (3)	0.011 (2)	0.005 (2)	0.002 (2)
C5	0.017 (3)	0.025 (3)	0.022 (3)	−0.001 (2)	0.004 (2)	0.001 (2)
C6	0.014 (5)	0.024 (6)	0.015 (5)	0.009 (5)	−0.001 (4)	0.001 (4)
C7	0.012 (4)	0.016 (4)	0.027 (4)	0.000 (3)	0.002 (3)	−0.003 (3)
C8	0.028 (3)	0.017 (2)	0.020 (3)	0.004 (2)	0.010 (2)	−0.001 (2)
C9	0.027 (3)	0.027 (3)	0.023 (3)	0.009 (3)	0.008 (3)	0.001 (2)
C10	0.021 (3)	0.026 (3)	0.019 (3)	0.007 (2)	0.008 (2)	0.003 (2)
N1	0.020 (5)	0.016 (5)	0.018 (4)	0.004 (4)	0.006 (4)	−0.009 (3)
N2	0.015 (5)	0.022 (5)	0.027 (5)	0.005 (4)	0.006 (4)	0.013 (4)
O1	0.028 (4)	0.023 (5)	0.019 (4)	0.010 (4)	0.003 (3)	−0.006 (4)
O2	0.024 (3)	0.023 (3)	0.029 (3)	−0.001 (2)	0.011 (2)	−0.001 (2)
O3	0.037 (2)	0.033 (2)	0.0169 (18)	0.0129 (18)	0.0092 (16)	0.0034 (16)
O4	0.035 (3)	0.038 (3)	0.021 (2)	0.022 (2)	0.0092 (19)	0.003 (2)
O5	0.018 (4)	0.013 (4)	0.031 (4)	0.000 (3)	0.010 (3)	0.002 (3)
O6	0.021 (3)	0.024 (3)	0.026 (3)	0.003 (2)	0.007 (2)	0.004 (2)
O7	0.031 (2)	0.035 (2)	0.0165 (18)	0.0136 (18)	0.0065 (16)	−0.0001 (16)
O8	0.035 (2)	0.048 (3)	0.020 (2)	0.023 (2)	0.0071 (17)	0.0018 (18)
Pd	0.01683 (17)	0.01537 (15)	0.01715 (16)	0.00487 (11)	0.00451 (11)	−0.00026 (10)

### Geometric parameters (Å, °)

**Table:**

C1—O2	1.231 (14)	C7—H11	1.0000
C1—O1	1.318 (14)	C8—C9	1.530 (7)
C1—C2	1.508 (17)	C8—H12	0.9900
C2—N1	1.483 (16)	C8—H13	0.9900
C2—C3	1.553 (11)	C9—C10	1.503 (8)
C2—H3	1.0000	C9—H14	0.9900
C3—C4	1.532 (7)	C9—H15	0.9900
C3—H4	0.9900	C10—O8	1.230 (7)
C3—H5	0.9900	C10—O7	1.323 (7)
C4—C5	1.485 (7)	Pd—N1	2.072 (9)
C4—H6	0.9900	N1—H2	0.85 (2)
C4—H7	0.9900	N1—H1	0.85 (2)
C5—O4	1.238 (7)	Pd—N2	2.005 (11)
C5—O3	1.316 (7)	N2—H10	0.85 (2)
C6—O6	1.243 (14)	N2—H9	0.85 (2)
C6—O5	1.289 (14)	Pd—O1	1.976 (8)
C6—C7	1.538 (16)	O3—H8	0.86 (2)
C7—N2	1.493 (16)	O5—Pd	2.024 (7)
C7—C8	1.529 (12)	O7—H16	0.85 (2)
			
O2—C1—O1	124.1 (11)	C9—C8—H12	109.1
O2—C1—C2	120.1 (10)	C7—C8—H13	109.1
O1—C1—C2	115.7 (11)	C9—C8—H13	109.1
N1—C2—C1	110.6 (9)	H12—C8—H13	107.8
N1—C2—C3	106.2 (8)	C10—C9—C8	111.8 (5)
C1—C2—C3	111.7 (9)	C10—C9—H14	109.3
N1—C2—H3	109.4	C8—C9—H14	109.3
C1—C2—H3	109.4	C10—C9—H15	109.3
C3—C2—H3	109.4	C8—C9—H15	109.3
C4—C3—C2	115.7 (5)	H14—C9—H15	107.9
C4—C3—H4	108.4	O8—C10—O7	123.0 (5)
C2—C3—H4	108.4	O8—C10—C9	122.2 (5)
C4—C3—H5	108.4	O7—C10—C9	114.8 (5)
C2—C3—H5	108.4	C2—N1—Pd	106.8 (8)
H4—C3—H5	107.4	C2—N1—H2	110 (6)
C5—C4—C3	114.6 (4)	Pd—N1—H2	108 (8)
C5—C4—H6	108.6	C2—N1—H1	119 (7)
C3—C4—H6	108.6	Pd—N1—H1	110 (7)
C5—C4—H7	108.6	H2—N1—H1	102 (9)
C3—C4—H7	108.6	C7—N2—Pd	106.3 (7)
H6—C4—H7	107.6	C7—N2—H10	105 (7)
O4—C5—O3	122.1 (5)	Pd—N2—H10	111 (9)
O4—C5—C4	123.5 (5)	C7—N2—H9	103 (7)
O3—C5—C4	114.5 (5)	Pd—N2—H9	114 (8)
O6—C6—O5	120.4 (11)	H10—N2—H9	115 (10)
O6—C6—C7	123.5 (10)	C1—O1—Pd	116.3 (8)
O5—C6—C7	116.1 (10)	C5—O3—H8	109 (5)
N2—C7—C8	114.0 (9)	C6—O5—Pd	113.1 (8)
N2—C7—C6	104.2 (10)	C10—O7—H16	117 (5)
C8—C7—C6	112.8 (8)	O1—Pd—N2	97.7 (4)
N2—C7—H11	108.6	O1—Pd—O5	178.6 (4)
C8—C7—H11	108.6	N2—Pd—O5	81.3 (4)
C6—C7—H11	108.6	O1—Pd—N1	81.7 (4)
C7—C8—C9	112.6 (6)	N2—Pd—N1	178.3 (6)
C7—C8—H12	109.1	O5—Pd—N1	99.3 (4)
			
O2—C1—C2—N1	161.2 (10)	C8—C9—C10—O7	−155.6 (5)
O1—C1—C2—N1	−20.8 (13)	C1—C2—N1—Pd	30.8 (10)
O2—C1—C2—C3	−80.7 (12)	C3—C2—N1—Pd	−90.6 (8)
O1—C1—C2—C3	97.3 (11)	C8—C7—N2—Pd	−168.6 (6)
N1—C2—C3—C4	−177.5 (6)	C6—C7—N2—Pd	−45.3 (9)
C1—C2—C3—C4	61.8 (10)	O2—C1—O1—Pd	176.4 (9)
C2—C3—C4—C5	74.0 (8)	C2—C1—O1—Pd	−1.5 (12)
C3—C4—C5—O4	−28.6 (8)	O6—C6—O5—Pd	171.2 (8)
C3—C4—C5—O3	151.5 (5)	C7—C6—O5—Pd	−10.6 (11)
O6—C6—C7—N2	−144.1 (10)	C1—O1—Pd—N2	−166.0 (8)
O5—C6—C7—N2	37.8 (12)	C1—O1—Pd—N1	15.7 (8)
O6—C6—C7—C8	−20.0 (15)	C7—N2—Pd—O1	−147.4 (7)
O5—C6—C7—C8	161.8 (8)	C7—N2—Pd—O5	33.7 (7)
N2—C7—C8—C9	−168.1 (8)	C6—O5—Pd—N2	−13.5 (7)
C6—C7—C8—C9	73.4 (10)	C6—O5—Pd—N1	164.8 (7)
C7—C8—C9—C10	−176.1 (6)	C2—N1—Pd—O1	−25.3 (7)
C8—C9—C10—O8	26.5 (8)	C2—N1—Pd—O5	153.7 (7)

### Hydrogen-bond geometry (Å, °)

**Table:**

*D*—H···*A*	*D*—H	H···*A*	*D*···*A*	*D*—H···*A*
N2—H9···O6^i^	0.85 (2)	2.15 (4)	2.979 (15)	162 (8)
N2—H10···O5^ii^	0.85 (2)	2.43 (8)	3.121 (13)	138 (10)
O7—H16···O4^iii^	0.85 (2)	1.80 (2)	2.639 (6)	169 (7)
O3—H8···O8^iv^	0.86 (2)	1.79 (3)	2.640 (6)	167 (7)
N1—H1···O2^v^	0.85 (2)	2.15 (3)	2.996 (15)	170 (9)
N1—H2···O1^vi^	0.85 (2)	2.30 (8)	2.998 (13)	139 (10)
N1—H2···O5^vii^	0.85 (2)	2.42 (7)	3.117 (13)	140 (10)

Symmetry codes: (i) *x*+1, *y*+1, *z*; (ii) *x*, *y*+1, *z*; (iii) *x*−1, *y*+1, *z*+1; (iv) *x*+1, *y*−1, *z*−1; (v) *x*−1, *y*−1, *z*; (vi) *x*, *y*−1, *z*; (vii) *x*+1, *y*, *z*.
